# Supplementary material for: Analysis of PPARγ Signaling Activity in Psoriasis
Source: Int J Mol Sci. 2021 Aug 10;22(16):8603. doi: 10.3390/ijms22168603 (PMC8395241; doi:10.3390/ijms22168603)
Supplement: Supplementary file 1 [file ijms-22-08603-s001.zip › Supplemental materials_Analysis of PPARg signaling activity in psoriasis/Pathway models/Models images and html files/PPARG inhibits phenotypes assotiated with psoriasis/28743.html]

thrombosis


# Disease thrombosis

|  |  |
| --- | --- |
| URN | urn:agi-meshdis:Thrombosis |
| Total Entities | 21 |
| Connectivity | 5147 |
| Name | thrombosis |

---

|  |  |
| --- | --- |
| ChildConcepts | cerebral thrombosis |
|  | middle cerebral artery thrombosis |
|  | brain thrombosis |
|  | thrombus |
|  | intracranial thrombosis |
|  | arterial thrombosis |
|  | blackfoot disease |
|  | catheter-related thrombosis |
|  | venous thrombosis |
|  | intracardiac thrombus |
|  | injection site thrombosis |
|  | leg vein thrombosis |
|  | microthrombosis |
|  | postoperative thrombosis |
|  | shunt thrombosis |
|  | experimental thrombosis |
|  | stent thrombosis |
|  | post-thrombotic syndrome |
|  | thromboangiitis obliterans |
|  | retinal vein thrombosis |
|  | prosthetic thrombosis |

---

|  |  |
| --- | --- |
| Pathway | Coagulation Cascade |
|  | Endothelial Cell Dysfunction in Pulmonary Hypertension |
|  | Antiphospholipid Antibodies in Endothelial Cell |
|  | Antiphospholipid Antibodies and Endothelial Cell Interaction |
|  | PPARG inhibits phenotypes assotiated with psoriasis |
|  | efects selected |
|  | Figure 2\_Levothyroxine and aspirin effects crossing |
|  | Figure 2\_Levothyroxine and aspirin effects crossing |
|  | 1\_Endothelial cells stimulate vasoconstriction and thrombosis in pulmonary hypertension |

---

|  |  |
| --- | --- |
| MedScan ID | 9010724 |

---

|  |  |
| --- | --- |
| Alias | Thrombotic Disorder |
|  | Thrombosis |
|  | thrombosis in acute |
|  | atherothrombosis |
|  | thrombosis of the arm |
|  | arm thrombosis |
|  | thrombo-obliterative diseases |
|  | acute thromboses |
|  | Thrombotic Disorders |
|  | rethrombosis |
|  | thrombotic disease |
|  | thromboses of the arm |
|  | thrombotic occlusions |
|  | thromboocclusive disease |
|  | thrombotic occlusion |
|  | re-thrombosis |
|  | thromboses in acute |
|  | thrombo-occlusive disease |
|  | rethromboses |
|  | atherothromboses |
|  | thrombo-obliterative disease |
|  | acute thrombosis |
|  | Thromboses |
|  | athero-thrombosis |
|  | thromboobliterative diseases |
|  | sclerothrombosis |
|  | thrombotic diseases |
|  | thrombotic |
|  | Blood Vessel Thrombosis |
|  | thromboobliterative disease |

---

|  |  |
| --- | --- |
| MeSH ID | D013927 |
|  | M0021435 |

---

|  |  |
| --- | --- |
| MeSH Heading | Thrombosis |

---
